# A Highly Selective Acetone Sensor Based on Coal-Based Carbon/MoO_2_ Nanohybrid Material

**DOI:** 10.3390/s24134320

**Published:** 2024-07-03

**Authors:** Min Zhang, Yi Han, Ting Liu, Hongguang Jia

**Affiliations:** 1 School of Physics Science and Technology, Xinjiang University, Urumqi 830046, China; hyzbdx2006@163.com (Y.H.); jiahg0802@163.com (H.J.); 2 College of Chemistry, Xinjiang University, Urumqi 830046, China; liut@xju.com

**Keywords:** coal, mixed molten salt, porous carbon, acetone sensors

## Abstract

High temperature represents a critical constraint in the development of gas sensors. Therefore, investigating gas sensors operating at room temperature holds significant practical importance. In this study, coal-based porous carbon (C-700) and coal-based C/MoO_2_ nanohybrid materials were synthesized using a simple one-step vapor deposition and sintering method, and their gas-sensing performance was investigated. The gas-sensing performance for several VOC gases (phenol, ethyl acetate, ethanol, acetone, triethylamine, and toluene) and a 95% RH high-humidity environment were tested. The results indicated that the C/MoO_2_-450 sample sintered at 450 °C exhibited excellent specific selectivity towards acetone at room temperature, with a response value of 4153.09% and response/recovery times of 10.8 s and 2.9 s, respectively. Furthermore, the C/MoO_2_-450 sample also demonstrated good repeatability and long-term stability. The sensing mechanism of the synthesized materials was also explored. The superior gas-sensing performance can be attributed to the synergistic effect between the porous carbon and MoO_2_ nanoparticles. Given the importance of enhancing the high-tech and high-value-added utilization of coal, this study provides a viable approach for utilizing coal-based carbon materials in detecting volatile organic compounds at room temperature.

## 1. Introduction

As a typical colorless VOC, acetone is closely related to our living and working environments. It is an essential raw material for organic synthesis, used to synthesize industrial products, including epoxy resins, polycarbonates, plexiglass, pharmaceuticals, and pesticides [1,2]. Moreover, it is a suitable solvent for smokeless gunpowder, celluloid, acetate, and paint [3,4,5,6]. Acetone is also used as a thinner, detergent, and extractant [7,8]. Additionally, acetone is a good breathing marker to distinguish healthy people from diabetics, as the acetone concentration in the exhaled air of healthy people ranges from 0.3 to 0.9 ppm. In contrast, the exhaled acetone concentration is higher than 1.8 ppm for people with diabetes [9]. Despite its wide range of applications in industry and medicine, acetone is a toxic, colorless, volatile gas that negatively affects the human central nervous system [10]. When inhaled acetone concentration exceeds 500 ppm, and it can irritate the eyes, nose, and throat. Headaches and dizziness may occur when acetone concentrations are above 1000 ppm. When the inhaled acetone concentration is high enough, the individual may lose consciousness, enter a coma, or even die. Therefore, it is crucial to conduct dynamic monitoring of acetone gas.

Coal is a high-quality carbon source for preparing carbon-based nanomaterials (carbon nanotubes, nanofibers, and graphene). It is widely used in supercapacitors, lithium–sodium batteries, and fuel cells due to its controllability, high abundance, and high chemical stability [11,12,13]; however, it is rarely applied in gas sensors. Coal is a polycrystalline material with a three-dimensional cross-linked network of aromatic and hydrogen units connected via bridge bonds and macromolecular structures [14]. Its structure possesses localized electrons and contains various functional groups and dangling bonds [15]. The cross-linked network structure enables rapid electron transfer. The functional groups and dangling bonds provide active sites for VOC gas adsorption, contribute to improve the gas-sensing performance [16]. According to the volatile substance content, coal is classified into lignite, bituminous coal, and anthracite [17]. Among them, the latter contains the highest carbon content and the least impurities with the highest degree of coalification. Therefore, we choose anthracite to prepare coal-based porous carbon materials.

MoO_2_ is an essential member of the transition metal oxide (TMO) family, with the commonality of transition metal oxides and unique metal-like conductivity [18]. In addition, it has strong structural stability due to its layered structure with gaps [19]; therefore, it is a promising battery anode, energy storage, and catalyst material. However, the aggregation of MoO_2_ nanoparticles limits its development and wide-spread application. Preparation of nanoscale sizes, composite formation, and porous-structure construction are effective ways to solve this agglomeration problem. Nanostructured molybdenum oxide has a larger specific surface area and unique nanostructure that ensures material integrity during electrochemical testing. Moreover, combining molybdenum oxide and the porous carbon phase through hybridization can create a synergistic effect between the two components [20]. The porous carbon in hybrid materials can prevent particle aggregation, stabilize nanomaterials’ structure, and maintain the stability of active substances [21,22,23].

In previous studies, metal oxide-based gas sensors have been widely welcomed due to their excellent performance. However, such materials often requires a high working temperature to achieve dynamic detection of target gases, meanwhile, traditional gas sensors based on these materials also exhibit poor selectivity [24],which greatly limits its application. Moreover, VOC gases would be inevitably encountered in industrial production and daily life, which not only harm the environment but also pose a threat to human health. In this study, we extensively selected some typical and common VOC gases: phenols (phenol), ketones (acetone), esters (ethyl acetate), alcohols (ethanol), amines (triethylamine), and aromatic hydrocarbons (toluene). In the gas sensing performance measurement, it was found that acetone was detected well and stably. In addition, we also considered the impact of humidity environment on sensor’s working. A 95% humidity environment was set in the performance measurement. Based on the above discussion, this study aims to explore room-temperature acetone sensors.

Therefore, nanohybrid materials were constructed based on coal-based porous carbon/MoO_2_ and applied for VOC gas detection. This study improved the technological content and economic added value of coal carbon resources and provided a feasible approach to using coal-based carbon materials in gas detection.

## 2. Experiments

### 2.1. Materials and Characterization

All reagents, except for coal, were used directly without any further purification. Coal was purchased commercially from a local market. Other chemicals used were lithium chloride (Shanghai Aladdin Biochemical Technology Co., Ltd., Shanghai, China); potassium chloride (Tianjin Xinbote Chemical Co., Ltd., Tianjin, China); ammonium molybdate tetrahydrate (NH_4_)_6_Mo_7_O_24_∙4H_2_O (Tianjin Xinbote Chemical Co., Ltd., Tianjin, China); H_2_SO_4_ (98%), HNO_3_ (63%), and HCl (37%) (Tianjin Shengmiao Fine Chemical Co., Ltd., Tianjin, China); sod absolute ethanol (Tianjin Huihang Chemical Co., Ltd., Tianjin, China), and ultrapure water H_2_O (self-made). The standard gases, such as phenol, acetone, ethyl acetate, ethanol, triethylamine, and toluene, were purchased from Sinopharm Group, (Sinopharm Chemical Reagent Co., Ltd., Shanghai, China).

Preparation of acidified coal: Coal was pretreated using a previously reported improved method [25]. First, the coal was pulverized to a certain degree. Then, 10 g of coal powder was carefully added into a flask containing 200 mL of mixed acid (V_HNO3_/V_H2SO4_ = 1:3) in an ice bath, which was stirred overnight. The above mixture was slowly added to 5 L distilled water, stirred and diluted until the foam disappeared, and then filtered with a Buchner funnel and washed with ultrapure water to neutral. Finally, a dark-brown powder was obtained after drying at 60 °C for 12 h.

Preparation of pure-phase coal-based porous carbon samples: Eutectic salt mixture KCl/LiCl (mass ratio of 11:9) was used as solvent and acidified coal was used as the carbon source. First, 0.2 g of acidified pulverized coal and 2.0 g of molten salt mixture were mixed and ground for 30 min to obtain precursors. Then, the precursor was placed in a porcelain boat and placed in a horizontal tube furnace, and nitrogen gas was introduced into the furnace tube after vacuuming. The temperature was raised at a rate of 3 °C min^−1^ and kept at 700 °C for 2 h. After the sample was naturally cooled, the molten salt was washed with HCI and deionized water, and the coal-based porous carbon sample, which was named C-700, was obtained after drying at 60 °C.

**Figure 1:**
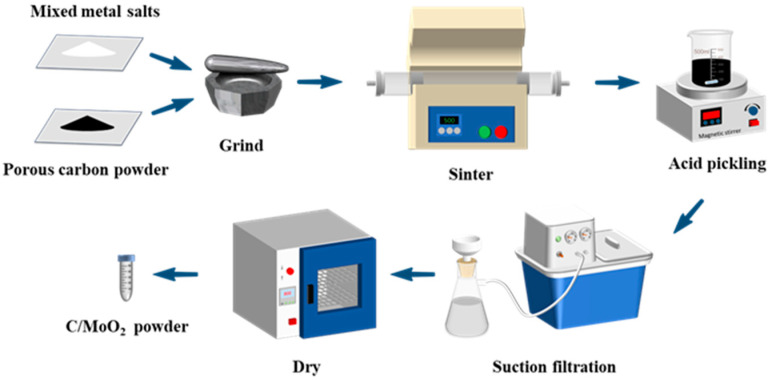
Preparation process of pure-phase and nanohybrid coal-based carbon materials.

Preparation of coal-based porous carbon/MoO_2_ nanohybrid samples: a eutectic salt mixture KCl/LiCl (mass ratio of 11:9) was used as the solvent and acidified coal was used as the carbon source. First, 0.1 g of acidified pulverized coal and 2.0 g of molten salt mixture were mixed. Then, (NH_4_)_6_Mo_7_O_24_∙4H_2_O (0.106 g) was added, and the precursor was obtained after grinding for 30 min. Subsequently, the precursor was placed in a porcelain boat, and then put it into a horizontal tube furnace, and the sintering temperature was set as 400–500 °C, and the temperature increased at a rate of 3 °C/min^−1^ for 0.5 h. After the sample was naturally cooled, the molten salt was washed with HCl and deionized water, and dried at 60 °C to obtain a coal-based porous carbon/MoO_2_ nanohybrid sample. The obtained hybrid samples were named as C/MoO_2_-400, C/MoO_2_-450, and C/MoO_2_-500 according to the different sintering temperatures. The above preparation process is shown in Figure 1.

The samples’ crystal structure and composition were determined by X-ray diffractometer (XRD, D8 advance, Bruker, Karlsruhe, Germany). The morphology of samples was observed by a field emission scanning electron microscope (SEM, Simgma 300, ZEISS, Oberkochen, Germany). The internal nanostructures and elemental distribution of samples were characterized by transmission electron microscopy (TEM, TecnaiG2 F20, FEI, Hillsboro, OR, USA). The relationship between mass and temperature of samples was analyzed by Thermogravimetry Analysis (TG, HITACHI STA7300, Hitachi, Tokyo, Japan). Raman Spectra (LabRAMHR800, HORIBA, Paris, France) is applied for detecting characteristic peaks of carbon. X-ray photoelectron spectroscopy is used for analyzing the elemental composition of samples (XPS, Thermo ESCALAB 250Xi, Waltham, MA, USA).

### 2.2. Fabrication of Sensors

**Figure 2:**
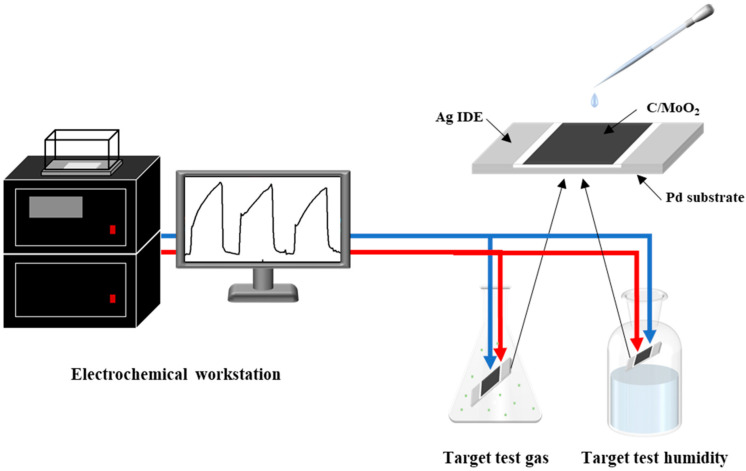
Gas-sensor manufacturing and performance testing.

An appropriate amount of prepared samples was added to a mortar. Then, deionized water was gradually added to the mortar with a mass ratio of 1:3 (coal-based porous carbon/MoO_2_ nanohybrid sample:H_2_O = 1:3, *w*/*w*). The mixture was ground to a fine powder and formed a paste. An appropriate amount of the paste was dipped with a brush and evenly coated on the Ag–Pt II electrode (Figure 2). This was dried at room temperature for 48 h and then electricity was applied to age the electrode plates. The external dimensions of the electrode sheet were 13.4 mm × 7 mm × 0.635 mm, with an interdigitated electrode width of 0.2 mm and a spacing of 0.2 mm.

### 2.3. Gas-Sensor Testing

According to Formula (1), different gas concentrations were prepared by the static gas-distribution method [26]. Q and V are the liquid volumes to be taken and the container volume for gas distribution, respectively. M is the amount of a gas molecule, d is the purity of the gas-distribution liquid, C is the configured gas concentration, and ρ is the density of liquid required for the configured gas. T_R_ and T_C_ are the ambient temperature and the temperature in the gas container, respectively. Since the experiments were conducted at room temperature, both T_R_ and T_C_ were at room temperature. To minimize the interference of relative humidity (RH), the specific preparation process of the target vapor was as follows: First, the volumetric flask was washed and dried. Second, the target liquid was injected into a volumetric flask, heated to produce the target vapor, and left to cool naturally at room temperature. Third, all devices were tested for gas sensing at 4 V.

(1) $$Q=\frac{V\times C\times M}{22.4\times D\times \rho }\times 10^{-9}(273+T_{R})/(273+T_{C})$$

Response = (I_g_ − I_0_)/I_0_ (2)

As shown in Figure 2, the photoelectric integrated test platform (CGS-MT) tested the sensor current at room temperature to record the current curve with time (I–T). The current response was defined as shown in Equation (2), where I_g_ and I_0_ are generally the sensor currents in the air and target gas. The response time is when the sensor reaches 90% of the time during which the current is stable after the target gas exposure, and the recovery time is the time taken for the current to change to 10% of the baseline current after the target gas is removed.

For the humidity tests involved in this study, a commercial electronic thermohygrometer (FY-12, Vanke Sheng, Shenzhen, China, 1–99% RH) was used to calibrate the pre-configured standard humidity volumetric flasks.

## 3. Results and Discussion

### 3.1. Characterization of Samples

**Figure 3:**
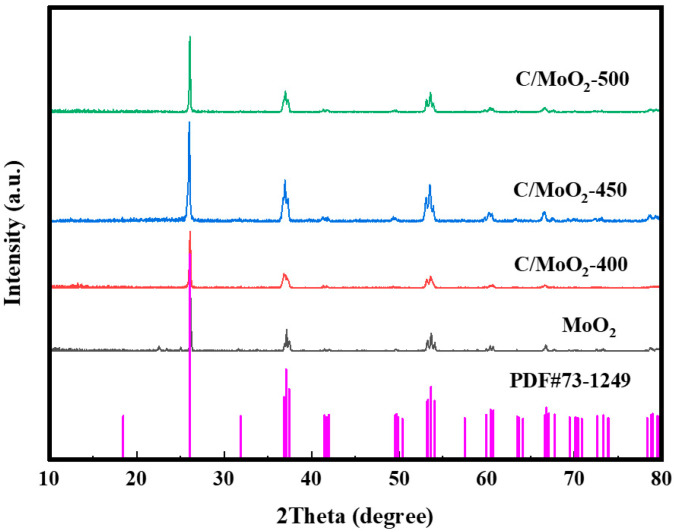
XRD spectra of C/MoO_2_-400, C/MoO_2_-450, C/MoO_2_-500, and MoO_2_ samples.

X-ray diffraction analysis (XRD) was performed first to characterize the samples’ phase composition. The XRD spectrum of samples calcined at different temperatures is shown in Figure 3. The prominent characteristic peaks of C/MoO_2_-400, C/MoO_2_-450, and C/MoO_2_-500, and single-component MoO_2_ samples, corresponded to MoO_2_ (JCPDS no. 73-1249); moreover, the diffraction peaks near 26°, 37°, 54°, and 60°, corresponded to crystal planes (110), (111), (−222), and (031) of MoO_2_, respectively, indicating the formation of MoO_2_ at all sintering temperatures and the successful synthesis of a single-component MoO_2_ sample. Since the carbon in C/MoO_2_-450 is amorphous, no characteristic carbon peaks were observed in XRD [27]. It is noteworthy that at the same diffraction angle, the C/MoO_2_ sample prepared at 450 °C exhibited stronger peak intensity compared with other samples, indicating a higher MoO_2_ content and better crystallinity. Therefore, the material prepared at this temperature theoretically possessed superior physicochemical properties, and the synergistic advantage between porous carbon and MoO_2_ was also more pronounced.

**Figure 4:**
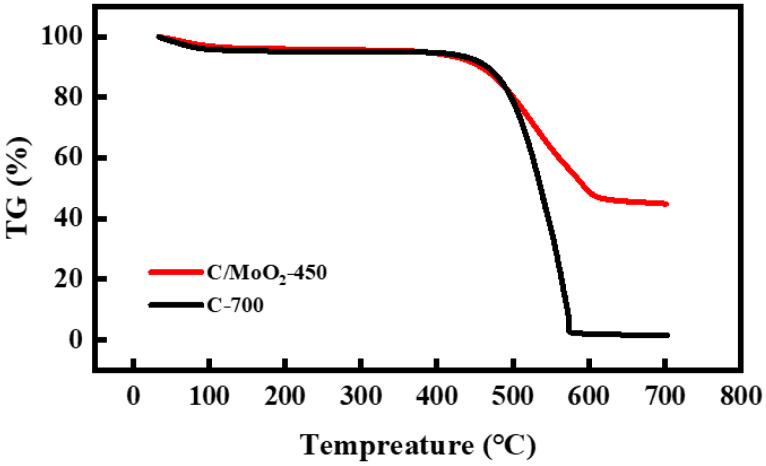
Thermogravimetric curves of C-700 and C/MoO_2_-450 samples.

To further validate this analytical result, we also analyzed the thermogravimetric curves of the samples in air. As shown in Figure 4, both the C-700 and C/MoO_2_-450 samples exhibited no significant thermogravimetric loss below 400 °C, indicating that the prepared coal-based porous carbon material possessed good structural stability at high temperatures. The thermogravimetric curve demonstrates a rapid downward trend between 450 °C and 600 °C, suggesting significant thermogravimetric loss in this temperature range, primarily attributable to the breakage of major chemical bonds in the carbon network (such as C–C bonds) and the pyrolysis of functional groups. This suggests that as the temperature increased, the stability of the sample decreased, resulting in significant weight loss when the temperature exceeded 450 °C. Therefore, combining the previous XRD test results, it can be determined that the optimal sintering temperature was 450 °C. Additionally, the thermogravimetric loss rate of the C/MoO_2_-450 sample was 55.1%, whereas the pure-phase C-700 sample exhibited a much higher loss rate of 98.5%. This remarkable difference in thermogravimetric loss further confirms the successful introduction of MoO_2_ into the C/MoO_2_-450 sample.

**Figure 5:**
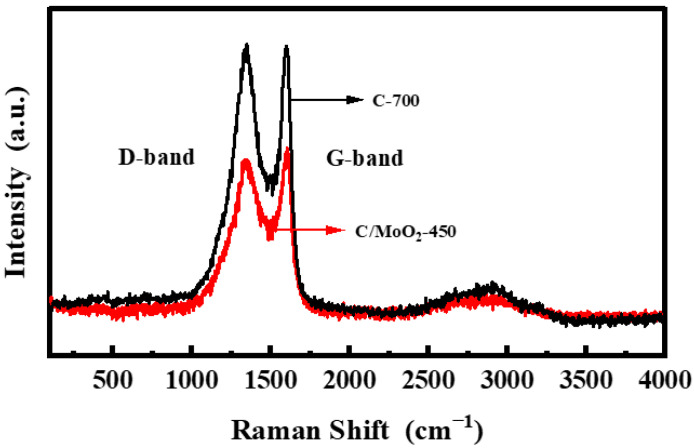
Raman spectra of C-700 and C/MoO_2_-450 samples.

Raman spectra were further employed to analyze the carbon materials present in the hybridized sample. The Raman spectrum of the C/MoO_2_-450 sample is shown in Figure 5. The G peak of the Raman spectrum corresponds to the first-order scattering of E_2g_ mode, representing the degree of graphitization in the ordered sp^2^ region. In contrast, the D peak represents disordered carbon, mainly including structural defects, amorphous carbon, and asymmetric structures at the edges. The spectrum of the C/MoO_2_-450 nanohybrid exhibits carbon characteristic peaks at 1348 cm^−1^ and 1604 cm^−1^, corresponding, respectively, to sp^3^ and sp^2^ hybridized atoms of disordered elements in the hexagonal graphite layer [28]. To quantitatively evaluate the graphitization degree and defect level of carbon materials, we introduced the parameter of the intensity ratio between the D peak and the G peak (I_D_/I_G_). Generally, a higher I_D_/I_G_ value indicates a lower graphitization degree of the carbon material, along with more significant structural defects [29]. In this analysis, the I_D_/I_G_ values of C-700 and C/MoO_2_-450 were 0.99 and 1.01, respectively. This indicates that the graphitization degree of C/MoO_2_-450 was significantly lower than that of the C-700 sample, while also forming more structural defects. These defects can provide a large number of active adsorption sites for gas adsorption, thus enhancing the sensitivity of its sensor.

**Figure 6:**
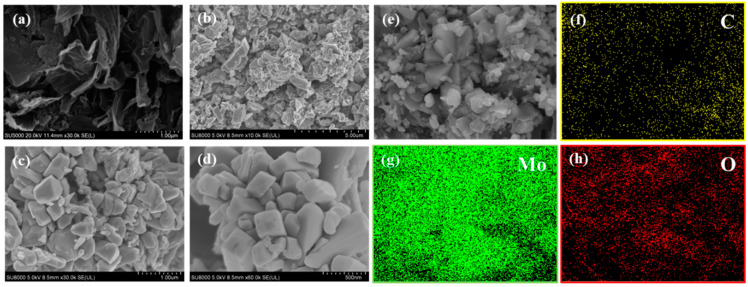
SEM images of all the samples. (**a**) SEM image of C-700, (**b**–**d**) SEM images of C/MoO_2_-450 with different magnifications, (**e**) the selected area for mapping of C/MoO_2_-450, and (**f**–**h**) the mapping graphs of different elements (C, Mo, O) in C/MoO_2_-450.

The morphology of pure-phase porous carbon C-700 and C/MoO_2_-450 samples was analyzed by scanning electron microscopy (SEM), transmission electron microscopy (TEM), and high-resolution TEM (HRTEM). The SEM of the pure-phase C-700 sample (Figure 6a) shows that it had a loose and porous structure. From the SEM of the C/MoO_2_-450 sample (Figure 6b–d), it can be seen that MoO_2_ particles with a size of approximately 50–80 nm were uniformly distributed in the loose and porous carbon network. Figure 6e–h display the mapping images of the C/MoO_2_-450 sample, providing intuitive information on the elemental distribution. Figure 6e selects a specific observation area, while Figure 6f–h correspond to the distribution maps of carbon, molybdenum, and oxygen elements, respectively. The benefits of the uniform distribution of these elements in the material are as follows: Firstly, the uniform distribution enables gas molecules to react fully with the sensitive material, thereby enhancing the sensitivity of the sensor. Secondly, the uniform distribution reduces the diffusion resistance of gas molecules within the material, allowing gases to diffuse faster to the sensitive regions, resulting in a faster response speed of the sensor. Through observing these images, we can clearly see that all elements in the sample exhibited a uniform distribution, and only C, Mo, and O were present, with no other impurity elements detected. This result fully demonstrates that the sample prepared in this experiment was composed solely of C and MoO_2_, with high purity and no contamination from other elements. Due to the addition of the mixed molten salt KCl/LiCl system, under high-temperature conditions, the liquid molten salt KCl/LiCl served as the reaction environment for ammonium molybdate tetrahydrate and acidified coal, enabling them to react in the form of “naked” ions, thereby forming smaller-sized MoO_2_ nanoparticles and uniformly dispersing them on the porous carbon. This unique loose and porous structure and the conductive carbon framework effectively increased the contact area between the material and gas molecules, while preventing the aggregation of MoO_2_ grains. These factors resulted in increased electron migration efficiency within the hybrid material, thereby effectively improving its electrical properties and potentially yielding a C/MoO_2_-450 nanohybrid material with enhanced detection performance.

**Figure 7:**
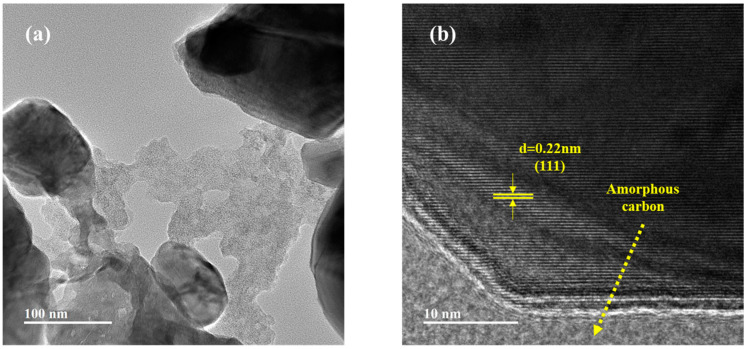
(**a**) TEM and (**b**) HRTEM images of C/MoO_2_-450 sample.

TEM images further confirm the formation of a hybrid structure. Figure 7a,b show the TEM and HRTEM images of the C/MoO_2_-450 sample. We can clearly observe the interconnection between C and MoO_2_, and this intertwined structure provides unique properties for the material. Meanwhile, the microporous structure on the carbon material that enhances the specific surface area is also evident. Figure 7b further exhibits a high-resolution transmission electron microscopy (HRTEM) image of the C/MoO_2_-450 sample. In this image, we can observe distinct lattice fringes, which are a direct reflection of the internal atomic arrangement of the material. Through precise measurements, it is found that the lattice fringe spacing of 0.22 nm corresponded to the (111) plane of MoO_2_. Additionally, we can clearly see the interface between MoO_2_ and amorphous carbon, indicating that MoO_2_ is embedded in the amorphous porous carbon network, forming a unique hybrid structure. This structure not only improved the stability of the material, but also facilitated the generation of synergistic effects between the two components.

**Figure 8:**
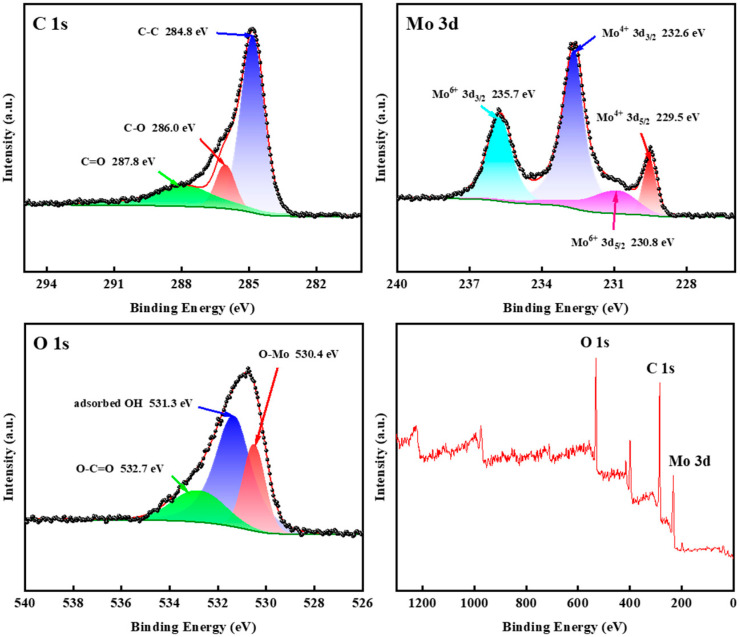
XPS spectra of C/MoO_2_-450sample.

X-ray photoelectron spectroscopy (XPS) analysis of the C/MoO_2_-450 sample is shown in Figure 8. XPS spectra suggest that C, Mo, and O elements existed in the sample. In the Mo 3d spectrum, the peaks corresponding to binding energies of 229.5 and 232.6 eV were originated from 3d_5/2_ and 3d_3/2,_ respectively_,_ for Mo^4+^; and the peaks at 230.8 and 235.7 eV were attributed to 3d_5/2_ and 3d_3/2_, respectively, for Mo^6+^ [30]. Mo^6+^ was due to the oxidation of the sample’s surface when exposed to air. In the C 1s spectrum, the characteristic peaks of 284.8 eV were attributed to C-C bands, while the peaks at 286.0 and 287.8 eV are orientated from C-O and C=O bonds [31]. Additionally, the binding energy of 530.4 eV corresponded to Mo-O in C/MoO_2_-450, while the peaks at 531.3 and 532.7 eV were attributed to -OH and O-C=O, respectively [30]. The XPS spectra further demonstrate that the molybdate was reduced by coal-based carbon during the calcination process, forming MoO_2_. The above characterization shows that the sample C/MoO_2_-450 was a nanohybrid composed of MoO_2_ and C.

**Figure 9:**
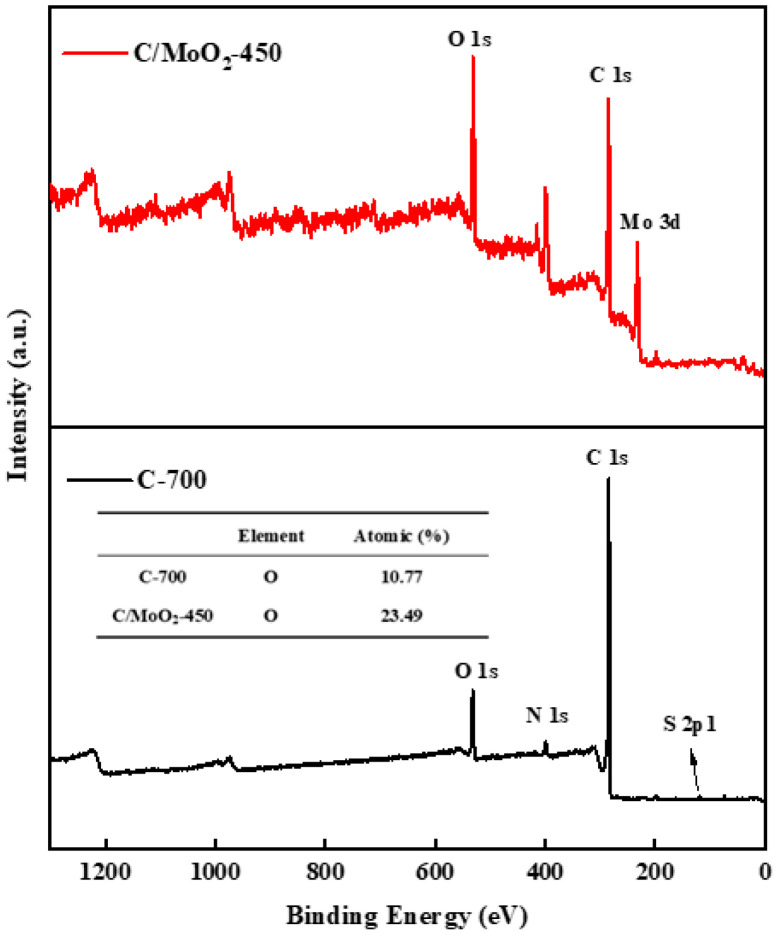
XPS spectrum wide scan of C/MoO_2_-450 and C-700 sample.

Upon comparing the XPS spectra of C-700 and C/MoO_2_-450 (Figure 9), it is evident that the O 1s peak intensity of the C/MoO_2_-450 hybrid sample exhibited an increase compared with the pure-phase coal-based porous carbon C-700. Detailed analysis revealed that the oxygen content within these samples was 10.77% for C-700 and 23.49% for the C/MoO_2_-450 hybrid. A change in oxygen content will directly affect the gas-sensing performance of the material: On the one hand, as the oxygen content increases, the material surface may form more oxygen ions or oxygen-adsorbed species, enabling rapid and highly sensitive detection of target gases [32]. On the other hand, an increase in oxygen content reduces the oxygen vacancies in the material, leading to an increase in the baseline resistance [33], which contributes to enhance the gas-sensing performance of the sensor material.

### 3.2. Gas-Sensing Performances

**Figure 10:**
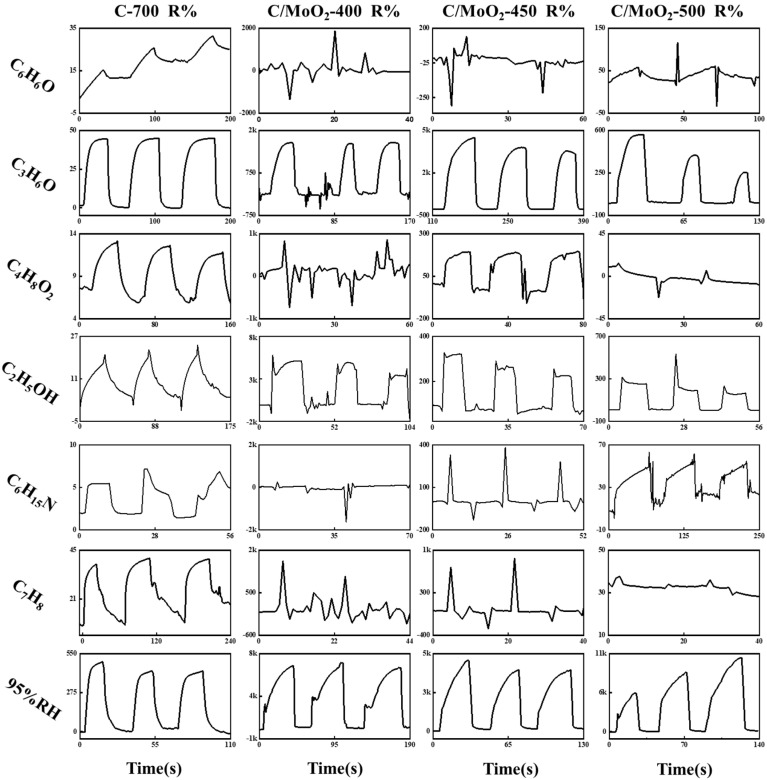
Response/recovery (R%) curve of C-700 and C/MoO_2_-400, C/MoO_2_-450, and C/MoO_2_-500 sensors for 1000 ppm phenol, acetone, ethyl acetate, ethanol, triethylamine, and toluene at room temperature.

Figure 10 shows the dynamic sensing properties of C-700, C/MoO_2_-400, C/MoO_2_-450, and C/MoO_2_-500 for phenol (C_6_H_6_O), acetone (C_3_H_6_O), ethyl acetate (C_4_H_8_O_2_), ethanol (C_2_H_6_O), triethylamine (C_6_H_15_N), toluene (C_7_H_8_), and the high-humidity environment (95% RH) at room temperature. The baseline corresponds to an environmental test curve of approximately 30% RH (relative air humidity). For sensing performance testing, sensors were employed in three consecutive cycles using a gas concentration of 10000 ppm. The current rose rapidly when the gas sensor was exposed to C_6_H_6_O, C_3_H_6_O, C_4_H_8_O_2_, C_2_H_6_O, C_6_H_15_N, and C_7_H_8_ and 95% humidity. In contrast, the current dropped rapidly and reached a steady state when the sensor was removed from the gas vapors and immediately exposed to air, reflecting the typical characteristics of n-type semiconductors. The pure-phase C-700 sensor showed a response of 42.62% to acetone, which was around an order of magnitude. The responses of nanohybrid C/MoO_2_-400, C/MoO_2_-450, and C/MoO_2_-500 samples to C_3_H_6_O were 1830.6%, 4153.09%, and 567.0%, respectively. Compared with pure-phase C-700, the response of composite sample sensors to C_3_H_6_O have been improved by one to three orders of magnitude. Among them, C/MoO_2_-450 exhibited the best response to 1000 ppm acetone, and its response to C_3_H_6_O have been improved by more than three orders of magnitude. The sensing performance has been significantly improved. Moreover, the performance of the C/MoO_2_-450 sensor was better than that of C/MoO_2_-400 and C/MoO_2_-500 sensors at room temperature. The responses of the C/MoO_2_-450 sensor to 1000 ppm C_6_H_6_O, C_4_H_8_O_2_, C_2_H_6_O, C_6_H_15_N, and C_7_H_8_ and 95% humidity were 0%, 188.1%, 322.4%, 0%, 0%, and 3953.1%, respectively, indicating the sensor’s good selectivity to acetone. The above results show that the C/MoO_2_-450 sensor possessed excellent acetone-detection ability. The dynamic response curves in Figure 10 in a humidity environment of 95% RH indicate that all C/MoO_2_ sensors had poor moisture interference resistance in high-humidity environments. In addition, after conducting gas-sensitivity tests on the synthesized pure MoO_2_ samples, it was found that they did not show a significant response to these six VOC gases mentioned in this study. This may have been due to the influence of material synthesis methods on their own structure and physicochemical properties [34]. In contrast, the C/MoO_2_-450 hybrid sample had a significant advantage, indicating that a hybrid material composed of coal-based carbon and MoO_2_ can significantly increase its gas sensitivity to acetone.

**Figure 11:**
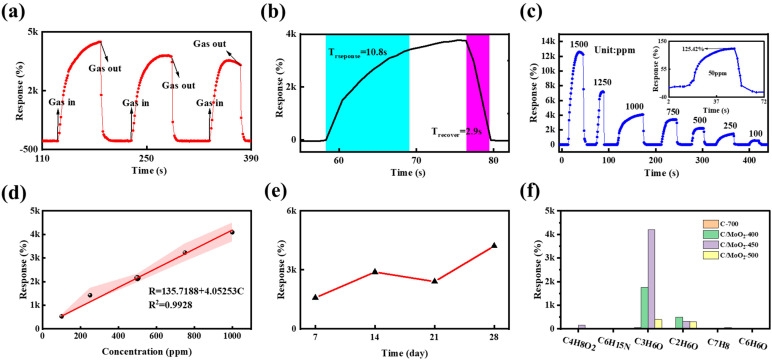
(**a**) Dynamic sensing curves of C/MoO_2_-450 in 1000 ppm acetone, (**b**) response/recovery time curve of C/MoO_2_-450 in 1000 ppm acetone atmosphere, (**c**) dynamic response curves of C/MoO_2_-450 for acetone from 50 to 1500 ppm, (**d**) linear fitting curves within 1000 ppm, (**e**) long term stability of C/MoO_2_-450 within one month, and (**f**) the selectivity of all sensors.

Gas-sensor repeatability is a crucial factor in practical gas-sensing applications. Figure 11a illustrates the repeatability of the C/MoO_2_-450 sensor for 1000 ppm acetone. The response and recovery curves remained constant after three cycles, suggesting that the sensor exhibited good reproducibility. Given gas-sensors’ performance requirements in practical applications, response/recovery times performance are also important. Figure 11b reflects that the response time of the C/MoO_2_-450 sensor was 10.8 s, and the recovery time was 2.9 s in a 1000 ppm acetone gas atmosphere, indicating the rapid response and recovery characteristics to acetone. Figure 11c shows the dynamic response curves of C/MoO_2_-450 for acetone in the concentration range of 50 to 1500 ppm. The sensor response increased with acetone concentration, and the increase in gas adsorption at the surface active site was the reason behind this phenomenon. Figure 11d shows the linear fitting curve for 1000 ppm acetone, and its linear relationship was as follows: R = 115.7188 + 4.0525C (R^2^ = 0.9928 in Figure 11d). Figure 11e shows the long-term stability of the C/MoO_2_-450 sensor over one month. In practical applications, selectivity is a key indicator since it suggests whether or not the sensor can counteract interference from other gases. At the same temperature and gas concentration, the response of the C/MoO_2_-450 sensor to acetone was much higher than that of other test gases (Figure 11f).

In Table 1, we compared the working temperature and response/recovery time of some acetone sensors reported in previous literature. It can be seen that the C/MoO_2_-450 sensor in this study showed excellent response/recovery time characteristics to acetone at room temperature, and could achieve rapid detection.

### 3.3. Gas-Sensing Mechanism

According to the previous description of the dynamic response curve, C/MoO_2_-450 exhibited n-type semiconductor properties. The gas-sensing mechanism of a typical n-type metal-oxide semiconductor is explained by the change in the thickness of the electron depletion layer caused by the redox reaction on the material’s surface (surface resistance control model) [41]. First, when the sensor is exposed to air at room temperature, oxygen molecules are adsorbed on the sensor surface, and the oxygen molecules trap electrons form the sample surface to form $O_{2\left(ads\right)}^{-}$. At this time, electrons are first captured from the conduction band of C/MoO_2_-450 due to the lack of electrons on the surface, forming an electron depletion layer. This results in a decrease in electron concentration, a thickening of the depletion layer, and a lower current state [40,42]. Subsequently, when the target gas reacts with oxygen ions, electrons are released back into the gas-sensing material, narrowing the electron depletion layer. Therefore, the material resistance is reduced and presents a high current state [43]. The above process can be described using the following equations [40,42].

(3) $$O_{2(gas)}↔O_{2(ads)}$$

(4) $$O_{2(ads)}+e^{-}\to O_{2\left(ads\right)}^{-}(T<100\;{}^{\circ}C)$$

(5) $$O_{2\left(ads\right)}^{-}+e^{-}\to 2O_{\left(ads\right)}^{-}(100\;{}^{\circ}C<T<300\;{}^{\circ}C)$$

(6) $$O_{\left(ads\right)}^{-}+e^{-}\to O_{\left(ads\right)}^{2-}(T>300\;{}^{\circ}C)$$

(7) $${CH}_{3}CO{CH}_{3}+4O_{2}^{-}\to 3{CO}_{2}+3H_{2}O+8e^{-}$$

**Figure 12:**
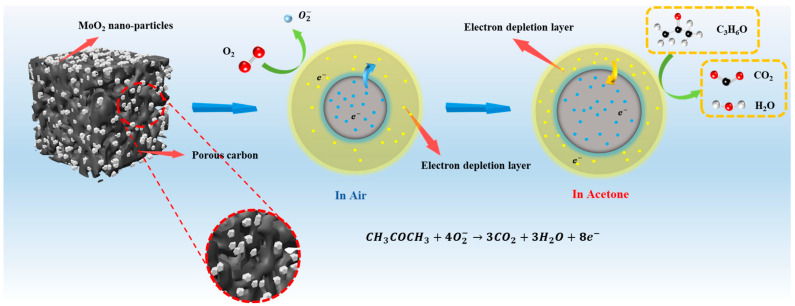
Schematic diagram of C/MoO_2_-450 sensing mechanism.

**Figure 13:**
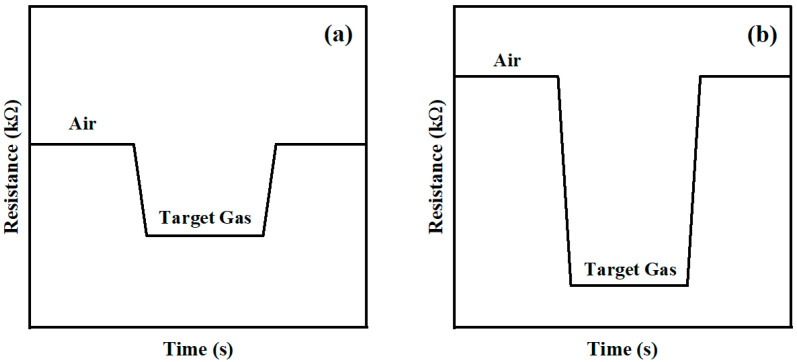
Resistance curves of (**a**) C-700 and (**b**) C/MoO_2_-450 samples.

As shown in Figure 12, the contact between MoO_2_ nanoparticles dispersed on porous carbon forms a homojunction potential barrier, increasing the height and width of the potential barrier, blocking the electron transportation and providing additional electrons for oxygen species on the sensing-material surface (Equation (4)). The potential barrier increases, meanwhile, according to the XPS results, the increase in oxygen content in the hybrid material leads to a reduction in oxygen vacancies, both of which, together, significantly increase the baseline resistance of the C/MoO_2_-450 sensor. When the sensing materials contact acetone vapor, the electrons released by the reaction with $O_{2\left(ads\right)}^{-}$ are transferred to C/MoO_2_-450, which reduces the sensing-materials’ barrier and further reduces gas-sensor resistance. The above discussion on the surface resistance control model and changes in the material’s barrier is summarized in the resistance curve image (Figure 13).

The sensing mechanism of the hybrid sample can also be explained from the perspective of material structure. The porous structure increases the contact area between the sensing material and the target gas. Meanwhile, the porous structure offers numerous active sites that can interact with target gas molecules, leading to more significant changes in the sensor’s output signal. On the other hand, the porous structure not only facilitates rapid gas diffusion and transport but also exhibits stress-dispersion effects, reducing material deformation and cracking, and the performance degradation of the sensor during long-term operation. In addition, MoO_2_ nanoparticles are uniformly distributed on porous carbon materials. The C/MoO_2_-450 hybrid exhibited a larger surface particle size (including more holes and wrinkles). The porous structure is conducive to attaching MoO_2_ nanocrystals to its surface, inhibiting the aggregation of MoO_2_ nanocrystals, and thus improving the structural stability of sensing materials. Due to its metal-like properties, MoO_2_ also has high conductivity [44], and the combination of the two significantly enhances the electron transport capacity in the sensing material. Moreover, the larger particle size of the C/MoO_2_-450 hybrid provides many adsorption sites to promote the adsorption and desorption of gas molecules [45]. The synergistic effect of uniformly distributed MoO_2_ nanoparticles and porous carbon structure results in a significant advantage of the obtained C/MoO_2_-450 over a single coal-based porous carbon in surface sensing.

## 4. Conclusions

Coal-based porous carbon (C-700) and coal-based C/MoO_2_ nanohybrid materials were synthesized by simple one-step vapor deposition and sintering methods, and their gas-sensing properties were studied. The results showed that the gas-sensing performance of the C/MoO_2_-450 hybrid sample with the introduction of a MoO_2_ component was significantly enhanced compared with the pure-phase coal-based porous carbon sample (C-700). The optimal performance was shown when the sintering temperature was 450 °C; among all VOC gases tested at room temperature, the C/MoO_2_-450 sensor showed the highest response (4153.09%) and specific selectivity to 1000 ppm acetone, while exhibiting fast response time (10.8 s) and recovery time (2.9 s), good response repeatability (over three measurements), and long-term stability (within one month). MoO_2_ nanoparticles positively impact coal-based porous carbon applications in gas sensors, especially for detecting VOC gases. This is mainly due to the synergistic effect between porous carbon and MoO_2_, and the gain effect from the homogeneous junction barrier between MoO_2_ nanoparticles. Therefore, combining MoO_2_ with coal-based porous carbon materials provides a potential and feasible method to improve gas sensitivity.

## Figures and Tables

**Table 1:** Comparison of response/recovery time of acetone sensors in different studies.

Sensing Materials	Acetone	Temperature	Response Time	Recovery Time	Reference
Cactus-like WO_3_–SnO_2_ nanocomposite	600 ppm	360 °C	14 s	16 s	[35]
Co_3_O_4_/ZnCo_2_O_4_ composite	100 ppm	255 °C	41 s	47 s	[36]
Fe_2_O_3_–CuO nanorod	100 ppm	240 °C	149 s	133 s	[37]
Bamboo raft-like Co_3_O_4_	200 ppm	180 °C	32 s	35 s	[38]
WO_3_ plate	200 ppm	307 °C	10 s	26 s	[39]
CdS-doped TiO_2_ nanocomposite	5000 ppm	Room temperature	55 s	115 s	[40]
C/MoO_2_ nanohybrid material	1000 ppm	Room temperature	10.8 s	2.9 s	This work

## Data Availability

Data are available upon request.
